# *N*,*N*′-Bis(4-chloro­phen­yl)urea

**DOI:** 10.1107/S1600536808011069

**Published:** 2008-04-26

**Authors:** Kong Mun Lo, Seik Weng Ng

**Affiliations:** a Department of Chemistry, University of Malaya, 50603 Kuala Lumpur, Malaysia

## Abstract

The carbonyl unit of the title compound, C_13_H_10_Cl_2_N_2_O, lies on a twofold rotation axis. The ring is aligned at 51.6 (1)° with respect to the N—C(=O)—N fragment. The two –NH– fragments of one mol­ecule form hydrogen bonds [2.845 (2) Å] to the C=O fragment of an adjacent mol­ecule, giving rise to the formation of a linear hydrogen-bonded chain.

## Related literature

For isostructural *N*,*N*′-bis­(4-bromo­phen­yl)urea, see: Lin *et al.* (2004 ▶). *N*,*N*′-Bis-(4-chloro­phen­yl)urea has been isolated as a co-crystal with a phthalazinium chloride; see: Wamhoff *et al.* (1994 ▶). For the self-condensation of 4-chloro­phenyl isocyanate to yield the title symmetrical urea, see: Fu *et al.* (2007 ▶); Jimenez Blanco *et al.* (1999 ▶).
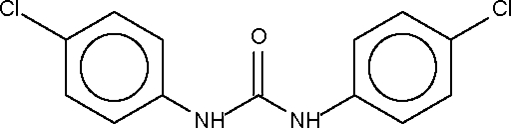


## Experimental

### 

#### Crystal data

C_13_H_10_Cl_2_N_2_O
*M* *_r_* = 281.13
Monoclinic, 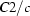

*a* = 27.093 (3) Å
*b* = 4.5768 (5) Å
*c* = 9.901 (1) Å
β = 96.389 (2)°
*V* = 1220.1 (2) Å^3^
*Z* = 4
Mo *K*α radiation
μ = 0.52 mm^−1^
*T* = 100 (2) K
0.20 × 0.20 × 0.10 mm

#### Data collection

Bruker SMART APEX diffractometer
Absorption correction: multi-scan (*SADABS*; Sheldrick, 1996 ▶) *T*_min_ = 0.862, *T*_max_ = 0.950
3703 measured reflections
1386 independent reflections
1210 reflections with *I* > 2σ(*I*)
*R*_int_ = 0.020

#### Refinement

*R*[*F* ^2^ > 2σ(*F* ^2^)] = 0.031
*wR*(*F* ^2^) = 0.096
*S* = 1.11
1386 reflections
87 parameters
1 restraint
H atoms treated by a mixture of independent and constrained refinement
Δρ_max_ = 0.31 e Å^−3^
Δρ_min_ = −0.29 e Å^−3^

### 

Data collection: *APEX2* (Bruker, 2007 ▶); cell refinement: *SAINT* (Bruker, 2007 ▶); data reduction: *SAINT*; program(s) used to solve structure: *SHELXS97* (Sheldrick, 2008 ▶); program(s) used to refine structure: *SHELXL97* (Sheldrick, 2008 ▶); molecular graphics: *X-SEED* (Barbour, 2001 ▶); software used to prepare material for publication: *publCIF* (Westrip, 2008 ▶).

## Supplementary Material

Crystal structure: contains datablocks global, I. DOI: 10.1107/S1600536808011069/tk2256sup1.cif

Structure factors: contains datablocks I. DOI: 10.1107/S1600536808011069/tk2256Isup2.hkl

Additional supplementary materials:  crystallographic information; 3D view; checkCIF report

## Figures and Tables

**Table 1:** Hydrogen-bond geometry (Å, °)

*D*—H⋯*A*	*D*—H	H⋯*A*	*D*⋯*A*	*D*—H⋯*A*
N1—H1⋯O1^i^	0.87 (1)	2.05 (1)	2.845 (2)	152 (2)

Symmetry code: (i) 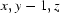.

**Fig. 1.**
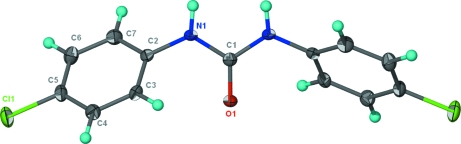
The molecular structure of (I) showing the atom-numbering scheme and 70% probability displacement ellipsoids. Hydrogen atoms are drawn as spheres of arbitrary radius. The unlablled atoms related by a 2-fold axis of symmetry.

## supplementary crystallographic information

### Comment

The title compound, a symmetrical urea derivative, was the unexpected product from the reaction of 4-chlorophenyl isocyanate with *p*-tolylsulfonic acid in ethanol. The carbonyl unit of (Cl-4-C_6_H_4_)NH–C(=O)–NH(C_6_H_4_-4-Cl) lies on a twofold rotation axis, Fig. 1, that relates one aromatic ring to the other. The ring is aligned at 51.6 (1) ° with respect to the N–C(=O)–N fragment. The two –NH– fragments of one molecule forms hydrogen bonds to the C=O fragment of an adjacent molecule, giving rise to the formation of a linear hydrogen-bonded chain (Table 1). The compound has previously been synthesized from the self-condensation of 4-chlorophenyl isocyanate in acetone (Fu *et al.*, 2007) and in water catalyzed by pyridine (Jimenez Blanco *et al.*, 1999).

### Experimental

4-Chlorophenyl isocyanate (1.0 g, 6.5 mmol) and *p*-toluenesulfonic acid (1.2 g, 6.5 mmol) were heated in ethanol (100 ml) for 1 h. The solution was filtered; evaporation of the solvent gave plates of the symmetrical urea.

### Refinement

Carbon-bound H-atoms were placed in calculated positions (C—H 0.95 Å) and were included in the refinement in the riding model approximation, with *U*(H) set to 1.2*U*_eq_(C).

The amino H-atom was located in a difference Fourier map, and was refined with a distance restraint of N–H 0.88±0.01 Å; its temperature factor was freely refined.

### Crystal data

**Table:**

C_13_H_10_Cl_2_N_2_O	*F*_000_ = 576
*M**_r_* = 281.13	*D*_x_ = 1.530 Mg m^−^^3^
Monoclinic, *C*2/*c*	Mo *K*α radiation λ = 0.71073 Å
Hall symbol: -C 2yc	Cell parameters from 1510 reflections
*a* = 27.093 (3) Å	θ = 3.0–28.2º
*b* = 4.5768 (5) Å	µ = 0.52 mm^−^^1^
*c* = 9.901 (1) Å	*T* = 100 (2) K
β = 96.389 (2)º	Block, colorless
*V* = 1220.1 (2) Å^3^	0.20 × 0.20 × 0.10 mm
*Z* = 4	

### Data collection

**Table:**

Bruker SMART APEXII diffractometer	1386 independent reflections
Radiation source: fine-focus sealed tube	1210 reflections with *I* > 2σ(*I*)
Monochromator: graphite	*R*_int_ = 0.020
*T* = 100(2) K	θ_max_ = 27.5º
ω scans	θ_min_ = 1.5º
Absorption correction: multi-scan(SADABS; Sheldrick, 1996)	*h* = −34→27
*T*_min_ = 0.862, *T*_max_ = 0.950	*k* = −5→5
3703 measured reflections	*l* = −10→12

### Refinement

**Table:**

Refinement on *F*^2^	Secondary atom site location: difference Fourier map
Least-squares matrix: full	Hydrogen site location: inferred from neighbouring sites
*R*[*F*^2^ > 2σ(*F*^2^)] = 0.031	H atoms treated by a mixture of independent and constrained refinement
*wR*(*F*^2^) = 0.096	*w* = 1/[σ^2^(*F*_o_^2^) + (0.0445*P*)^2^ + 1.607*P*] where *P* = (*F*_o_^2^ + 2*F*_c_^2^)/3
*S* = 1.11	(Δ/σ)_max_ < 0.001
1386 reflections	Δρ_max_ = 0.31 e Å^−^^3^
87 parameters	Δρ_min_ = −0.29 e Å^−^^3^
1 restraint	Extinction correction: none
Primary atom site location: structure-invariant direct methods	

### Fractional atomic coordinates and isotropic or equivalent isotropic displacement parameters (Å^2^)

**Table:**

	*x*	*y*	*z*	*U*_iso_*/*U*_eq_	
Cl1	0.293344 (15)	0.99597 (10)	0.33207 (4)	0.02417 (17)	
O1	0.5000	0.9101 (4)	0.7500	0.0163 (4)	
N1	0.46380 (5)	0.4789 (3)	0.67795 (15)	0.0149 (3)	
H1	0.4640 (8)	0.292 (2)	0.691 (2)	0.024 (5)*	
C1	0.5000	0.6399 (5)	0.7500	0.0130 (4)	
C2	0.42311 (6)	0.6073 (3)	0.59591 (15)	0.0131 (3)	
C3	0.43093 (6)	0.8150 (4)	0.49760 (16)	0.0152 (3)	
H3	0.4638	0.8730	0.4854	0.018*	
C4	0.39102 (6)	0.9373 (4)	0.41754 (17)	0.0175 (4)	
H4	0.3963	1.0823	0.3520	0.021*	
C5	0.34334 (6)	0.8455 (4)	0.43438 (16)	0.0162 (3)	
C6	0.33491 (6)	0.6357 (4)	0.52957 (17)	0.0183 (4)	
H6	0.3021	0.5729	0.5391	0.022*	
C7	0.37498 (6)	0.5180 (4)	0.61096 (17)	0.0176 (4)	
H7	0.3695	0.3754	0.6774	0.021*	

### Atomic displacement parameters (Å^2^)

**Table:**

	*U*^11^	*U*^22^	*U*^33^	*U*^12^	*U*^13^	*U*^23^
Cl1	0.0159 (2)	0.0299 (3)	0.0249 (3)	0.00200 (17)	−0.00584 (17)	0.00510 (17)
O1	0.0173 (8)	0.0094 (8)	0.0207 (8)	0.000	−0.0037 (6)	0.000
N1	0.0146 (7)	0.0082 (6)	0.0208 (7)	−0.0002 (5)	−0.0028 (6)	0.0007 (5)
C1	0.0128 (10)	0.0123 (11)	0.0141 (10)	0.000	0.0025 (8)	0.000
C2	0.0141 (7)	0.0104 (7)	0.0142 (7)	0.0001 (6)	−0.0006 (6)	−0.0027 (6)
C3	0.0124 (7)	0.0162 (8)	0.0167 (8)	−0.0028 (6)	0.0004 (6)	−0.0013 (6)
C4	0.0184 (8)	0.0178 (8)	0.0159 (8)	−0.0013 (6)	−0.0001 (6)	0.0016 (6)
C5	0.0138 (8)	0.0189 (8)	0.0152 (8)	0.0018 (6)	−0.0023 (6)	−0.0017 (6)
C6	0.0118 (8)	0.0233 (9)	0.0199 (8)	−0.0016 (6)	0.0024 (6)	−0.0009 (7)
C7	0.0176 (8)	0.0174 (8)	0.0178 (8)	−0.0019 (6)	0.0026 (6)	0.0026 (6)

### Geometric parameters (Å, °)

**Table:**

Cl1—C5	1.741 (2)	C3—C4	1.386 (2)
O1—C1	1.237 (3)	C3—H3	0.9500
N1—C1	1.363 (2)	C4—C5	1.386 (2)
N1—C2	1.422 (2)	C4—H4	0.9500
N1—H1	0.87 (1)	C5—C6	1.382 (2)
C1—N1^i^	1.363 (2)	C6—C7	1.387 (2)
C2—C7	1.390 (2)	C6—H6	0.9500
C2—C3	1.393 (2)	C7—H7	0.9500
			
C1—N1—C2	122.9 (1)	C5—C4—C3	119.2 (2)
C1—N1—H1	118 (1)	C5—C4—H4	120.4
C2—N1—H1	119 (1)	C3—C4—H4	120.4
O1—C1—N1	122.7 (1)	C4—C5—C6	121.3 (2)
O1—C1—N1^i^	122.7 (1)	C4—C5—Cl1	119.0 (1)
N1—C1—N1^i^	114.6 (2)	C6—C5—Cl1	119.65 (13)
C7—C2—C3	119.5 (2)	C7—C6—C5	119.21 (15)
C7—C2—N1	119.6 (1)	C7—C6—H6	120.4
C3—C2—N1	120.8 (1)	C5—C6—H6	120.4
C4—C3—C2	120.4 (2)	C6—C7—C2	120.42 (15)
C4—C3—H3	119.8	C6—C7—H7	119.8
C2—C3—H3	119.8	C2—C7—H7	119.8
			
C2—N1—C1—O1	0.4 (2)	C3—C4—C5—C6	0.3 (3)
C2—N1—C1—N1^i^	−179.6 (2)	C3—C4—C5—Cl1	−179.1 (1)
C1—N1—C2—C7	−129.4 (2)	C4—C5—C6—C7	0.9 (3)
C1—N1—C2—C3	52.6 (2)	Cl1—C5—C6—C7	−179.8 (1)
C7—C2—C3—C4	1.6 (2)	C5—C6—C7—C2	−0.8 (3)
N1—C2—C3—C4	179.6 (2)	C3—C2—C7—C6	−0.5 (2)
C2—C3—C4—C5	−1.5 (2)	N1—C2—C7—C6	−178.5 (2)

Symmetry codes: (i) −*x*+1, *y*, −*z*+3/2.

### Hydrogen-bond geometry (Å, °)

**Table:**

*D*—H···*A*	*D*—H	H···*A*	*D*···*A*	*D*—H···*A*
N1—H1···O1^ii^	0.87 (1)	2.05 (1)	2.845 (2)	152 (2)

Symmetry codes: (ii) *x*, *y*−1, *z*.

## References

[bb1] Barbour, L. J. (2001). *J. Supramol. Chem.* **1**, 189–191.

[bb2] Bruker (2007). *APEX2* and *SAINT* Bruker AXS Inc., Madison, Wisconsin, USA.

[bb3] Fu, J.-L., Wang, Z. & Zhu, H. (2007). *Huaxue Shiji*, **29**, 187–188.

[bb4] Jimenez Blanco, J. L., Saitz Barria, C., Benito, J. M., Ortiz Mellet, C., Fuentes, J., Santoyo-Gonzalez, F. & Garcia Fernandez, J. (1999). *Synthesis*, pp. 1907–1914.

[bb5] Lin, Q., Zhang, Y.-M., Wei, T.-B. & Wang, H. (2004). *Acta Cryst.* E**60**, o696–o698.

[bb6] Sheldrick, G. M. (1996). *SADABS* University of Göttingen, Germany.

[bb7] Sheldrick, G. M. (2008). *Acta Cryst.* A**64**, 112–122. 10.1107/S0108767307043930 18156677

[bb8] Wamhoff, H., Bamberg, C., Hermann, S. & Nieger, M. (1994). *J. Org. Chem.* **59**, 3985–3993.

[bb9] Westrip, S. P. (2008). *publCIF* In preparation.

